# Advances in Hydrogel Polymers for Microbial Control in Water Systems

**DOI:** 10.3390/polym16152205

**Published:** 2024-08-02

**Authors:** Adenike A. Akinsemolu, Helen Onyeaka

**Affiliations:** 1 Department of Integrated Science, Adeyemi Federal University of Education, Ondo 351101, Ondo, Nigeria; 2 School of Chemical Engineering, University of Birmingham, Birmingham B15 2TT, UK

**Keywords:** hydrogel polymers, microbial control, pathogens, microorganisms, antimicrobial

## Abstract

Every year, contaminated water is responsible for over one million deaths globally. Microbiology leads other fields in the development of solutions to water contamination to reduce these deaths while advancing the achievement of SDG 6, which aims to ensure universal access to water and sanitation. This article explores hydrogel polymers as a solution to water contamination through microbial control. Using a systematic approach, this study collects, reviews, analyzes, and synthesizes the findings of studies on the structure, properties, and mechanisms used by hydrogel polymers in pathogen control in water systems, emphasizing recent advances in microbiology that have improved the antimicrobial properties of hydrogel polymers, enhanced their synthetic properties, and improved their overall ability to control the spread of pathogens in water. Other additional notable findings, including the applications of hydrogel polymers in water systems, the environmental implications of using the method to decontaminate and purify water for various purposes, and the regulatory standards needed to reinforce the viability and effectiveness of the adaptation of hydrogel polymers for the control of harmful or unwanted microorganisms in water systems, inform the presented inferences on the future of hydrogel technologies and new opportunities for the expansion of their commercial use.

## 1. Introduction

According to the World Health Organization (WHO), half of the global population lacks access to clean and safe drinking water [1]. Consequently, they use dirty and contaminated water, which is associated with a high incidence of preventable diseases. The WHO links contaminated water with at least 1.4 million preventable deaths every year, namely owing to diarrhea, cholera, and other diseases that are spread through contaminated water [1]. In 2021, diarrhea alone caused 829,000 deaths globally, including 300,000 deaths of children below the age of five [2]. These deaths could have been prevented if the affected families and communities had access to clean water for drinking, sanitation, and hand hygiene. The main causes of water contamination include agricultural chemicals, chemicals from household and industrial waste, sewage, fuels, pharmaceutical waste, and microorganisms [3]. Harmful microorganisms enter water sources through sewage containing human fecal matter, waste from hospitals and pharmaceutical companies, agricultural waste containing animal waste, and industrial waste [4]. The most common microorganisms associated with water contamination are *Escherichia coli*, *Shigella dysenteriae*, *Shigella flexneri*, *Shigella boydii*, *Shigella sonnei*, *Salmonella enterica*, and *Vibrio cholerae*, which are responsible for diseases such as cholera, dysentery, acute diarrhea, gastroenteritis, and typhoid [5]. The death toll from these diseases and their impact on the quality of life of affected individuals, households, communities, nations, and regions warrant the development and implementation of microbial control approaches to reduce water contamination by disease-causing pathogens.

Hydrogel polymers present an effective, environmentally friendly, and affordable method of microbial control in contaminated water [6]. Hydrogel polymers are insoluble polymers that are made from either natural or synthetic polymers and can hold large volumes of water without dissolving [7]. Their ability to absorb large volumes of water makes hydrogel polymers suitable for various applications, including the treatment of contaminated or polluted water. The application of polymers in wastewater treatment harnesses their adsorption abilities, through which contaminants adhere to their surface to remove them from the contaminated water [8]. Currently, hydrogel polymers are used to remove both organic and inorganic contaminants such as metal ions and dyes from polluted water. They can be adapted to remove microbial contaminants as well as to leverage microorganisms’ adherence to hydrogel surfaces.

Recent and past reviews on the topic have explored various dimensions of the adaptation of hydrogel polymers for the control of harmful or unwanted microorganisms in water systems. A recent study explored the application of hydrogel polymers to the treatment of industrial effluent, focusing on applications such as dye removal, the adsorption of heavy metal ions, and the viability of different types of hydrogels for the treatment of industrial waste [9]. However, the study does not address hydrogels with antimicrobial abilities or the integrations of remediating microorganisms with hydrogel polymers to remediate microbial pollutants. A second study evaluated the use of natural hydrogel polymers with antimicrobial abilities in the remediation of environmental pollutants [10]. One of the applications explored in the study is the use of hydrogels in water treatment, highlighting mechanisms such as adsorption, the use of hydrogels as carriers of remediating microorganisms, and the use of hydrogels with natural antimicrobial abilities. While the study highlights these mechanisms, it does not identify the hydrogel polymers with antimicrobial abilities nor does it discuss their adaptation for different water systems. A third study explores the use of cellulose-based hydrogels in the treatment of wastewater [11]. Its focus is on the remediation of metal ions and its discussion of wastewater remediation relative to the use of microorganisms is limited to an identification of the environmental conditions affecting the stability and performance of cellulose hydrogels. Evidently, most studies on the use of hydrogel polymers in water treatment focus on other pollutants such as metal ions and fail to address microbial pollution comprehensively. Furthermore, the integration of antimicrobials with hydrogel polymers as well as polymers with natural antimicrobial properties and their application in controlling microbial pollution in the treatment of water are not addressed comprehensively in recent studies and reviews. To this end, this article explores how historic and recent advances in hydrogel polymers can and are being adapted to control microorganisms in water systems with the overarching goal of evaluating the efficiency, cost-effectiveness, sustainability, and replicability of their use for microbial control in water systems.

## 2. Materials and Methods

A broad search was conducted using the keywords and prompts “hydrogel polymers”, “the applications of hydrogel polymers”, “hydrogel polymers and water treatment”, “hydrogel polymers and microbial control”, and hydrogel polymers and microbial control in water systems”. Relevant research studies, case studies, and books were identified, schemed through, and screened using inclusive criteria that isolated peer-reviewed sources, factual case studies describing relevant applications of hydrogel polymers, and recent publications published between 2010 and 2024 for relevance and timeliness. The research articles, studies, books, and case studies that met the inclusion criteria were studied extensively and analyzed and information was extracted for use in this study.

## 3. Results

### 3.1. Fundamentals of Hydrogel Polymers

**Figure 1:**
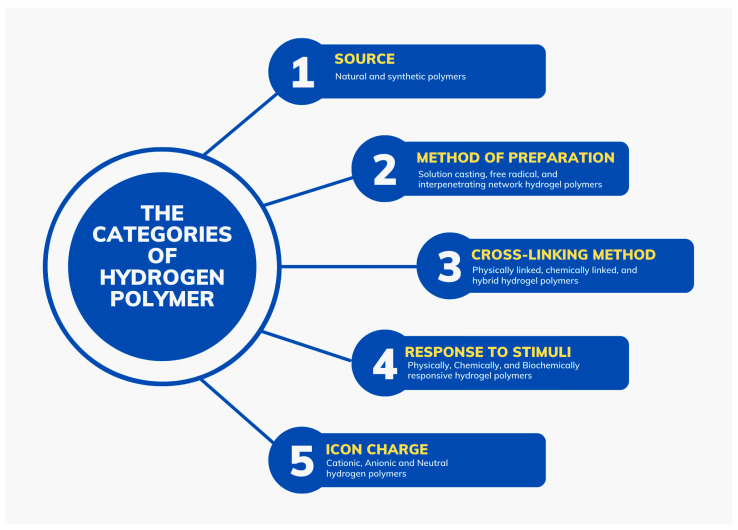
The categories of hydrogel polymers.

Hydrogel polymers, also known as polymeric hydrogels, are polymeric materials with hydrophilic qualities and structures that enhance their ability to hold large volumes of water [7]. The polymers have porous cross-linked three-dimensional structures that can be synthesized from one or more monomers. They are classified into five categories as shown in Figure 1. The first group of polymers, which are categorized by source, include natural and synthetic polymers. Natural hydrogel polymers are formulated from materials extracted from nature, such as chitosan and cellulose, while synthetic hydrogel polymers are formulated from petroleum and include poly (hydroxyethyl methacryla te) PHEMA and polyacrylic acid (PAA) [12,13]. The second classification characterizes hydrogel polymers by their method of preparation, yielding polymers produced through solution casting, free radical mechanism, and interpenetrating networks [14]. Third, the cross-linking method used in the formulation of hydrogel polymers is used to categorize them into chemical and physical cross-linked polymers based on the type of reaction used in their formulation. Due to the differences in these reactions, chemically cross-linked hydrogels have permanent links while their physically linked counterparts have temporary connections [13]. Fourth, hydrogel polymers exhibit different responses to physical or chemical stimuli such as pH, temperature, and magnetic and electric fields. Finally, hydrogel polymers fall under three distinct categories based on network electrical charge, which determines their ability to swell and shrink. These are neutral, cationic, and anionic hydrogel polymers [13].

Due to their significant hydrophilic qualities, hydrogel polymers can hold water within their interstices without affecting their network structure [14]. Furthermore, since the polymers can be synthesized from one or more monomers, their ability to hold water can be modified and both their physicochemical and mechanical properties enhanced [10]. The properties that make hydrogels suitable for water purification include their porosity, hydrophilicity, separation capability, switchable wettability, and the presence of different states of water in their structure [15]. Hydrogel polymers have pores or interstices through which they absorb water. This quality of the polymers can be manipulated to improve their ability to absorb higher amounts of liquid [16]. Hydrophilicity refers to the ability of polymeric hydrogels to absorb water and moisture. The hydrogels are adapted for water absorption by their crosslinks, which prevent them from dissolving in water. Third, the water absorbed by the polymers can exist in three states, namely polymer-bound water, intermediate water, and free water. The polymers’ ability to absorb and store some of these states such as free water can be enhanced by increasing porosity. Additionally, the storage of intermediate water requires less energy while the mechanisms involved in the absorption and presence of bulk water enable the concentration of reactive agents such as bactericides [15]. Finally, switchable wettability allows hydrogel polymers to release absorbed water, enhancing their recyclability. These qualities make hydrogel polymers effective water purifiers and decontaminants.

Some hydrogels have natural microbial abilities that naturally adapt them for microbial control in wastewater. These hydrogels are classified based on the microorganisms against which they possess antimicrobial activity. The first category of hydrogel polymers with antimicrobial properties is antibacterial polymers, which gain their inherent antibacterial qualities from the materials used in their formulation. These materials include antibacterial peptides, chitosan, and organic acids, which are both biocompatible and biodegradable [17]. Some of the polymers that fall under this category target bacterial strains that have developed resistance against other antimicrobial agents. For instance, polymers with crude ethyl acetate extract (CEE) are particularly effective against Methicillin-resistant Staphylococcus aureus (MRSA) and other multi-drug resistant strains of bacteria [18]. A second category of polymers have natural antifungal properties that enable them to kill fungi on contact. These include hydrogels such as amphogel, which has desirable qualities such as prompt action against fungi, reusability, and biocompatibility, Poly (hexamethylene biguanide) hydrochloride (PHMB), and poly-ε-lysine (ε-PL) [19]. These hydrogels, together with others without antimicrobial properties, have been used successfully for the remediation of wastewater, particularly in the control of microbial contaminants. Some of the hydrogels that have been used for this purpose include macroporous antimicrobial polymeric gel (MAPG), polyacrylamide (PAAm) hydrogels, and polyethylene glycol (PEG) [20]. See Figure 1.

### 3.2. Synthesis of Hydrogel Polymers for Microbial Control

Polymeric hydrogels are synthesized through chemical, physical, and hybrid bonding through various routes, including solution polymerization, interpenetrating network formation, solution casting and mixing, the radiation method, and free radical mechanism [14]. Solution polymerization yields highly porous gels using a monomer solution, a chemical initiator, and a cross-linking agent [21]. The initiator is activated using a redox initiator system, temperature, or UV radiation. Solution polymerization is ideal for the large-scale production of hydrogel polymers for various purposes, including water treatment and decontamination, due to its low cost, fast production of polymers, and the significant capacity of the resulting hydrogel polymer to absorb water [22]). Comparatively, free radical polymerization, a type of chain growth polymerization, is regarded as the most suitable technique for the synthesis of hydrogels based on amides, acrylates, and other monomers. It produces hydrogels from natural polymers, yielding polymerized polymers that do not allow impurities to pass through. Consequently, polymers produced through the synthesis pathway are ideal for microbial control in water systems as well as the removal of other impurities from water [22]. Like hydrogels produced through solution polymerization, the products of free radical polymerization are formed using crosslinking agents, initiators, and monomers. A third method of hydrogel preparation uses ionizing high-energy radiation such as electron beams and gamma rays to form cross-linked polymers [7]. The polymers have a significantly high absorption capacity, their particle size and porosity can be adjusted to meet desired levels, and their re-wetting capability is adequate. Furthermore, the polymers are durable and stable in various stages of absorption and adsorption and are broken down easily upon disposal since they are biodegradable. These qualities, which make the polymers easily adaptable for microbial control in contaminated water, have led to their extensive application in the biomedical field [23]. Ultimately, the synthesis method used to produce hydrogel polymers influences the viability of their use in controlling microbial contamination in water by having direct implications on their cost, porosity, stability during water absorption and storage, and structural integrity.

The applications of hydrogel polymers beyond water decontamination and microbial control in water systems include tissue engineering, drug delivery, and the production of medical dressing, biosensors, and contact lenses [14]. The magnitude of these uses has necessitated the dedication of research, funding, and resources to synthesis techniques to yield new applications, enhance the ability of hydrogel polymers to carry out existing uses, reduce the cost of their production, and improve their recyclability and biodegradability to make them more environmentally friendly. These efforts and resources have yielded several advances in synthesis techniques. One such advancement is the emergence of 3D bioprinting technology, which has made the customization of hydrogel polymers to specific uses relatively easier [24]. A second advancement in the synthesis of hydrogel polymers is the combination of cross-linked polymeric hydrogel (HDG) networks with nanoparticles to yield polymerized hydrogels with high water content and flexibility [25]. This has been found to enhance their mechanical properties and make them more suitable for specific applications such as water decontamination. These and other advances in the synthesis of polymers enhance their usability and applicability to the treatment and purification of contaminated water. However, these uses of hydrogel polymers raise some challenges. For instance, regardless of the synthetic pathway followed in their creation, hydrogels based on synthetic monomers lack antimicrobial activity [26]. This limits their applicability for microbial control in water systems and raises the need for the consideration of natural monomers in the synthesis of polymerized hydrogels for use in water treatment. Second, some synthesis methods are not environmentally friendly and use hazardous solvents [26]. This raises the need for greener synthesis approaches that use less energy and rely on less hazardous solvents. Third, some synthesis methods are not cost-effective and require specialized equipment [14]. This raises the issue of the economic viability of the use of hydrogel polymers produced using these methods for microbial control.

### 3.3. Mechanisms of Microbial Control by Hydrogel Polymers

Hydrogel polymers use various techniques to purify water and remove pathogenic contaminants in dirty water or wastewater. First, they erect barriers that encapsulate and immobilize microorganisms. Hydrogels are the ideal material for the encapsulation of living cells [27]. Traditionally, this ability was used to protect the encapsulated living cells, such as bacteria and other microorganisms, from damage during their delivery into the body as drugs to fight pathogens. The ability has since been adapted for the removal of harmful microorganisms from water systems using the same mechanisms. Essentially, the polymers’ high moisture and water content provide an ideal environment for microorganisms to thrive [28]. Once the microorganisms are bound to the polymers, the polymers’ cross-linked nature encapsulates and immobilizes them, controlling their spread until the proper conditions for their release are achieved. The mechanism used by hydrogel polymers in encapsulation and immobilization mimics the natural biofilm formed by microorganisms. In addition to attracting microorganisms, holding them using cross-linked polymer networks, and serving as separation media to draw them from contaminated water, hydrogels can inhibit microbial growth by depriving microorganisms of nutrients. Finally, some hydrogel polymers produce antimicrobial agents that eliminate microorganisms. An example of such polymers is chitosan-based hydrogels, which contain antiviral, antibacterial, and antifungal qualities. Antimicrobial hydrogel polymers use various mechanisms to kill the microorganisms against which they are deployed. Some damage the membrane of the microorganisms and release the dead microbes to prevent their accumulation in the gel [29]. This solves a critical problem of the retention of dead microorganisms by hydrogel polymers, freeing them up for reuse. Others use electrical stimulation moderated by external stimuli such as light and heat to release high concentrations of active oxygen, which kills microorganisms such as bacteria [17]. A third category of antimicrobial physical hydrogels, particularly those formulated through physical crosslinking with hyaluronic acid, form hydroxyl free radicals that destroy the cells of bacteria, effectively killing them [30]. Notably, some antimicrobial hydrogel polymers have selective properties and can target specific species of microorganisms. This solves the problem of the elimination of good and beneficial microorganisms alongside pathogenic and potentially invasive microorganisms that would upset biodiversity in the water bodies to which wastewater flows after treatment. To demonstrate the antimicrobial efficacy of hydrogel polymers, the diameter of inhibition zones for various hydrogel polymers tested against different bacterial strains is shown in Table 1 below.

### 3.4. Applications of Hydrogel Polymers in Water Systems

**Figure 2:**
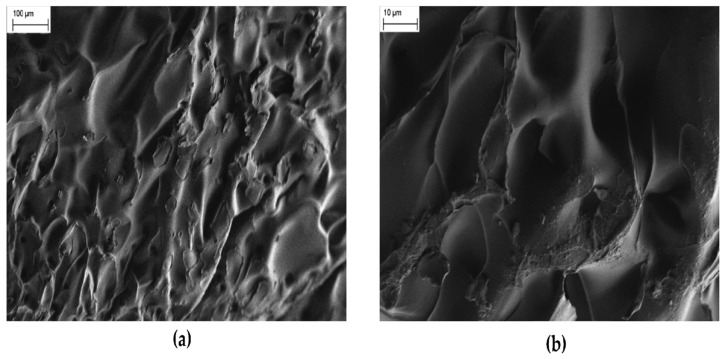
(**a**) Field emission scanning electron microscopy (FESEM) image of a pure PNIPAM hydrogel. (**b**) FESEM image of the same pure PNIPAM hydrogel at higher magnification [52].

Hydrogel polymers are used as adsorbent materials for the purification of drinking water. Chitosan-based hydrogels are particularly preferred for the absorption of contaminants to produce quality drinking water due to their desirable qualities. They include the sustainability of the polymers since chitosan is a natural polymer that is biodegradable and has low levels of toxicity, the antiviral, antifungal, and antimicrobial qualities of chitosan, which enable the polymers to target, encapsulate, and eliminate specific microorganisms, and the low cost of production of chitosan-based polymers, which makes their use in drinking water system affordable and economical [41]. For a detailed summary of the characteristics, mechanisms, and applications of hydrogel polymers, see Table 2. Another common trend in microbial control in drinking water systems, in addition to the use of biodegradable polymers with antimicrobial qualities such as chitosan, is the use of a combination of renewable sources of energy together with natural hydrogel polymers to purify water contaminated with pathogens [42]. One such hydrogel polymer was developed by researchers at Princeton University in 2023 [43]. The researchers developed a solar absorber gel that uses sunlight to eliminate water contaminants, including bacteria. The water purification system uses a hydrogel made from poly(N-isopropylacrylamide) or PNIPAm to absorb and release water at set temperatures. A FESEM image of the polymer is shown in Figure 2 below. When temperatures fall below 33 degrees Celsius or 91 degrees Fahrenheit, the hydrogel absorbs water from its designated source of water, which can be any water body including rivers and lakes. Once the gel is taken out of the water and heated by sunlight to temperatures above 33 degrees Celsius, it begins to release the water while filtering pollutants and contaminants such as microorganisms, microplastics, and heavy metals [43]. The gel has been tested on large water videos and the large-scale production of clean drinking water researchers are currently working to develop a smaller size model for use in households. A similar hydrogel polymer developed to purify water contaminated with microplastics, oil, metals, and small molecules was found to be effective in the removal of bacteria, particularly Escherichia coli, from contaminated water as well [44]. The hydrogel uses solar energy to purify water. It eliminates bacteria by leveraging their adhesive capabilities to attach them to their surface and the generation of a surface layer to contain the microorganisms upon the release of the now-purified water [44].

In addition to their application in the purification of drinking water, hydrogel polymers are used extensively in the treatment of wastewater, particularly in the removal of hard metals, dyes, and other organic and inorganic pollutants. The removal of these pollutants is primarily performed through adsorption by leveraging various interactions between the polymers and the pollutants or contaminants they target. Some of the common interactions used to eliminate these unwanted substances in wastewater include hydrophobic interactions, which prevent the gels from dissolving in water, hydrophilic interactions through which the hydrogel polymers absorb water, chelation, through which the ions of pollutant metals are bonded to the polymers, and ion exchange [15]. These interactions have been adapted to the use of polymers produced from various organic and inorganic materials in wastewater treatment. For instance, a polyacrylic acid hydrogel polymer, a synthetic and high-molecular-weight polymer, has been found to be highly effective in wastewater treatment. The polymer has most of the suitable qualities, including a high capacity for adsorption and reusability, which enhances the polymer’s sustainability for the application, cost-effectiveness since the polymer is cheap and easy to produce, and rapid removal of kinetics [6]. The hydrogel polymer has been found to be effective in the treatment of industrial wastewater. One recent development in the adaptation of hydrogel polymers for wastewater treatment is the use of cellulose-based polymers in water decontamination systems. The polymers are low-cost and biodegradable and their adsorption capabilities can be enhanced [45]. They have been successfully integrated into the treatment of wastewater in hybrid water systems [45]. The typical wastewater treatment process is a five-step process. It begins with the pretreatment stage, in which particles and dirt are bound to form larger particles that settle at the bottom of the wastewater. In the second stage, further coagulation and flocculation occur. Polymer flocculants are introduced at this stage to separate more particles from the water before the third stage [15]. The integration of hydrogel polymers begins in the third stage, in which purification occurs. In this stage, hydrogel polymers are incorporated to remove pollutants and contaminants such as pathogens, hard metals, and dyes in wastewater through adsorption before the water proceeds to the second-last stage of tertiary treatment [46]. In the fourth stage, oxidation, membrane filtration, and other chemical processes occur before the water is directed to clarifiers as the last step in its treatment. Therefore, hydrogel polymers are integrated into the secondary treatment stage of wastewater treatment, preceding tertiary treatment to complete a series of physical, chemical, and mechanical processes that rid the water of solid and microscopic contaminants and pollutants.

The applications of hydrogel polymers in both drinking water systems and wastewater treatment are enhanced and scaled for use in industrial water systems. While evaluating the applicability and effectiveness of the polymers in the treatment of industrial effluent, Flores-Valenzuela, Gonzalez-Fernandez, and Carranza-Oropeza identified some of the factors considered before their integration in industrial water treatment plants, which include their regeneration costs, maintenance needs, and capacity for adsorption [9]. In industrial water systems, hydrogels play more than one role. In addition to the treatment of wastewater and sludge using the five-step process described above, the polymers are adapted for use in cooling systems such as industrial cooling towers. This application leverages their hydrophilic and hydrophobic properties, which are part of the polymers’ self-adaptive cooling systems. The two abilities allow them to lose water through evaporation in high temperatures and regenerate when temperatures fall, creating a cooling effect during the day or in instances of high heat [47]. In addition to their integration into industrial cooling systems and their role in the treatment of industrial effluent, hydrogel polymers are used widely in the food, pharmaceutical, and agriculture industries [48]. In the food industry, their ability to absorb water is leveraged and adapted for the development of food packaging that controls humidity and microbial activity [49]. In the pharmaceutical industry, hydrogels are used in drug delivery, wound dressing, and wound management, which leverage their ability to absorb moisture to keep wounds dry [50]. In agriculture, hydrogel polymers enhance the capacity of the soil to hold water in addition to serving as nutrient conduits and reservoirs [51]. All these applications leverage the ability of hydrogel polymers to absorb, hold, and release water, which are the same qualities upon which their application in drinking water purification, wastewater treatment, and the treatment of industrial effluent are founded.

**Table 2:** Overview of hydrogel polymer characteristics, mechanisms, and applications in water microbial control.

	Description	Applications	Examples	Reference
**Absorption Capacity**	Ability to hold large volumes of water without dissolving, due to porous cross-linked structures.	Water purification, removal of organic and inorganic contaminants	Polyacrylamide (PAAm), Polyethylene glycol (PEG)	[20]
**Synthesis Methods**	Includes solution polymerization, free radical polymerization, and radiation methods.	Tailoring properties for specific water treatment needs	Solution polymerization for large-scale water decontamination	[14]
**Mechanisms of Action**	Encapsulation and immobilization of microorganisms, release of antimicrobial agents.	Controlling spread of pathogens in drinking and wastewater systems	Chitosan-based hydrogels for their antiviral and antibacterial properties	[27,28,31,32]
**Environmental Impact**	Biodegradability, toxicity, and impact on non-target organisms.	Ensuring sustainability and safety in water treatment	Biodegradable chitosan versus synthetic hydrogels	[14,24,31,32]
**Technological Advances**	Smart hydrogels responsive to environmental changes, integration with nanotechnology.	Enhancing effectiveness and application range in water systems	Smart hydrogels for adaptive water purification systems	[48,52,53]

## 4. Discussion

### 4.1. Performance Evaluation and Optimization

A comparison of the effectiveness of hydrogel polymers with other methods of microbial water treatment sets the polymers apart as one of the most effective means of controlling microorganisms in water systems. Other methods of microbial control in water systems include boiling, filtration, chlorination, sedimentation, and coagulation [54]. While effective in microbial control in drinking water systems, chlorination requires supplementation with other methods such as boiling as traces of pathogens have been isolated in chlorinated water [55]. Similarly, traces of microorganisms such as *Bacillus* spp. have been found to survive boiling, particularly if an open container is used for the process [53,56]. Filtration, on the other hand, is highly effective, removing nearly all traces of harmful microorganisms such as *Coliforms bacteria*, *Clostridium perfringens*, and *Enterococcus faecalis* [57]. However, the method is costly, has a high potential for toxicity to both human beings and the environment, and is incompatible with some water systems [58]. The drawbacks of these methods of microbial control in water systems warrant an evaluation of the effectiveness of hydrogel polymers, a viable alternative, general water treatment and purification, the environmental impact of their use, and the potential for integration with current and emerging technologies to enhance their effectiveness.

The fundamental property of hydrogel polymers is their hydrophilic nature and ability to hold large volumes of water [59]. This property, together with its characteristics, synthetic routes, mechanisms for microbial control, and applications in water treatment has been discussed extensively in the literature. These parameters can be used to evaluate their effectiveness in controlling microorganisms to eliminate pathogens in drinking water, wastewater, and industrial effluent. One of the most effective methods to this end is the Water Quality Index, which evaluates the quality of drinking water using parameters established by drinking water standards [60]. One of these parameters is the absence of microorganisms in the water [60]. This method could be applied by determining the species and population of microorganisms in water before its treatment or purification using hydrogel polymers. The drinking water, wastewater, or industrial effluent would be tested again for microorganisms after its purification to determine the effectiveness of the polymers as well as the quality and safety of the water for its intended purpose. A second method focuses on the activities of the polymers rather than the outcome of their applications. Typically, hydrogel polymers have inherent qualities that make them suitable for microbial control. For instance, chitosan, a natural polymer that is biodegradable and has low levels of toxicity, produces hydrogels with antiviral, antifungal, and antimicrobial qualities that enable them to target, encapsulate, and eliminate specific microorganisms [41]. Such abilities enhance the effectiveness of the polymers that possess them in microbial control. Third, the mechanical interactions between the cells of microorganisms and polymeric substances are good indicators of the effectiveness of hydrogel polymers in microbial control. For instance, the stiffness of hydrogels is a key determinant of their ability to encapsulate microorganisms [61]. However, the characteristics of the microorganisms, such as their ability to adhere to the surfaces of the hydrogel polymers, also influence encapsulation and affect the effectiveness of the gels in microbial control. Fortunately, the qualities of hydrogel polymers can be enhanced and adapted for the control of different microorganisms.

While the qualities and characteristics of both polymers and microorganisms are crucial determinants of the effectiveness of the hydrogels in microbial control, some parameters beyond both elements influence the performance of hydrogels as well as the outcome of their application. First, polymer composition determines the degree of adherence of microorganisms to hydrogel polymers. Polymer coating, for instance, affects the adherence of microorganisms such as bacteria [62]. The resulting differences in the level of adhesion to hydrogel polymers affect their ability to remove pathogens from drinking or wastewater. Another dimension of polymer composition is the material used in their formulation. Some polymers are made from materials that have natural antimicrobial qualities, which make them highly effective in microbial control while the rest rely on other mechanisms to remove microorganisms and other contaminants from water. Hydrogels that contain chitosan, for instance, have natural antimicrobial properties. Inorganic hydrogels can also be enhanced to contain antimicrobial qualities through their augmentation with nanoparticles that damage the cells of microorganisms [63]. Second, the properties of individual polymers influence their ability to eliminate or control microorganisms in water systems. The properties of hydrogel polymers differ based on the material from which they are produced and their adaptable characteristics, such as porosity. These characteristics determine the performance and effectiveness of the polymers. For instance, the hydrophilic structure of hydrogels is an indicator of their capacity to hold water, which influences their ability to treat and purify water by volume. Similarly, their foundational material, which is either natural or synthetic, influences their mechanical strength, durability, and rate of degradation, which affect both their performance and the length of time for which they can be used and reused in water treatment [7]. Third, the environmental conditions under which hydrogel polymers are used for microbial control in water systems influence their performance. Different polymeric hydrogels have different optimum levels of performance at different conditions such as temperature. Furthermore, they demonstrate different responses to chemical stimuli such as pH, physical stimuli such as light, and biological stimuli such as enzymes [64]. Therefore, these conditions will influence the performance of the polymers and aid in the determination of the suitability of different polymers for different environmental conditions. These determinants of the performance of hydrogel polymers can be used to optimize it in different water systems. Organic hydrogels can be augmented with inorganic elements in their networks to enhance durability and reusability, reduce costs, and simplify their production [15]. Second, hydrogel polymers can be adapted for different environmental conditions to enhance their performance and increase their effectiveness in microbial control. This can be conducted simply through the use of materials that are suitable for the conditions of different water systems or the adoption of smart hydrogel technology, which produces hydrogels that can respond to and adapt to environmental stimuli [65]. Finally, the integration of hydrogel polymers with existing water treatment systems optimizes their effectiveness in different water systems. A proposed solution to water turbidity demonstrates this strategy. The proposed strategy involves incorporating hydrogel polymers in set chambers in municipal water supply systems to purify water as it flows through the pipes to households and commercial premises. Integrating the polymers with existing water systems is inexpensive since it relies on existing systems.

### 4.2. Environmental and Health Impacts

Whilst hydrogel polymers are effective in the elimination of microorganisms from water, inexpensive, and environmentally friendly, particularly when the polymers used are organic and biodegradable, their use raises the issues of toxicity and the potential impacts on non-target organisms. Hydrogels that are synthesized from natural polymers are non-toxic [14]. However, some hydrogel polymers, particularly those that are synthesized from synthetic polymers, are toxic, cause extensive damage to the environment, and are incompatible with live cells. This raises the dilemma of the safety of their use to remove microbial and other contaminants from water, particularly drinking water. On the one hand, they are highly effective in microbial control, durable and reusable, easily and cheaply produced, and effective in the removal of other harmful and toxic contaminants such as metal ions from water. Furthermore, their properties can be enhanced and adapted to specific environmental conditions; but, on the other hand, they could be toxic [66]. Fortunately, their toxicity can be addressed by changing the crosslinkers used in their synthesis, which are often the source of toxicity [67]. A second challenge that arises in the use of hydrogel polymers in microbial control is their elimination of all microorganisms, including beneficial microbes. This can be addressed through the augmentation of hydrogel polymers with specific microorganism recognition elements that bind select microorganisms to their surface without capturing other microorganisms [68]. However, this could have some implications on the cost, effectiveness, and ease of production of the polymers.

In addition to the inherent lack of selectivity relative to microorganisms and the toxicity of some hydrogel polymers, their use in microbial control in water systems raises the issues of environmental impact. Natural polymers are naturally biodegradable and have little impact on their environment during use and upon disposal. However, synthetic polymers, which are often more effective than their organic counterparts, can be non-biodegradable [69]. Furthermore, they are made from petroleum, whose production and use are detrimental to the environment. Fortunately, some conditions can be established to degrade synthetic hydrogels. These include the use of microorganisms such as fungi to degrade the polymers [69]. This addresses the problem of the environmental impact of hydrogel polymers and justifies their continued use in microbial control in water systems. However, their use requires the establishment of and adherence to strict regulatory and health safety standards. These include restrictions on the types and materials of hydrogels that can be used to treat drinking water, water quality indices to determine the level of purification reached by the polymers, and the maximum levels of toxicity above which polymers cannot be used in water treatment or purification.

### 4.3. Future Perspectives and Innovations

Hydrogel technology is evolving and advancing to yield new applications of hydrogels, improve the effectiveness of existing applications, and improve the synthesis of hydrogels to make them more suitable for both types of applications. One of the emerging trends in hydrogel technology is smart hydrogels. The hydrogels are responsive to stimuli and environmental conditions such as temperature, pH, and light [70]. Their responsiveness to these triggers is reversible such that they can adapt quickly to changes in their environment and go back to their initial form just as promptly. They adapt by changing their shape, through interactions with solvents such as water, and via their hydrophilicity. In the pharmaceutical industry, these qualities of smart hydrogels are used to control dosage and dosing frequency, ensure the drug is only delivered to target tissues by minimizing its accumulation in non-target tissues, and prolong the drug release period [70]. In water systems, the qualities of smart hydrogels can be used to adapt them to changes in conditions such as acidity in wastewater, temperature in industrial water treatment plants, and pressure from piped water systems, to which they can respond by changing their shape. The viability of the use of smart hydrogels in water treatment has been evaluated, validated, and found to yield significant benefits, including easy synthesis, biocompatibility, and low costs [6]. A second emerging trend in hydrogel technology is responsive systems. These can be a feature of smart hydrogels. The gels can swell in high temperatures and contract in cold temperatures while others respond to light, pressure, and molecules such as glucose [71]. Some of the advantages of responsive hydrogels include fixed timing and precision [71]. These qualities enhance their performance in the treatment and purification of water by allowing the setting of specific timelines for their application. Once determined, timelines can be set automatically to determine the optimum purification. A third emerging trend, which is particularly relevant to microbial control, is the integration of sensing techniques in hydrogel polymer synthesis to allow them to capture microorganisms. This innovation has been adapted for the successful capture and elimination of bacteria [69]. Fourth, hydrogel polymers are being integrated with other technologies to enhance their efficacy in microbial control in water systems and other applications. Their integration with nanotechnology, for instance, yields nanohydrogels, which have superior benefits such as biodegradability, selective abilities, and a wide range of applications [72]. These emerging trends and technologies yield highly effective hydrogel polymers with improved attributes, which can be commercialized for use in the treatment of water across a variety of water systems. However, they raise the challenges of cost and the need for specialized knowledge and equipment.

## 5. Conclusions

In conclusion, hydrogel polymers are networks of polymers with hydrophilic qualities. Their properties, which include porosity, the ability to absorb large volumes of water without dissolving, and their ability to swell allow the polymer’s adaptation for the treatment of water through the removal of contaminants, including pathogens and unwanted microorganisms. Some hydrogel polymers have additional properties that are leveraged for microbial control in water systems such as antimicrobial abilities. Other qualities that enhance the overall performance of the polymers in water treatment and purification include biodegradability, compatibility with live cells, sensitivity to environmental stimuli, and mechanical strength. The use of hydrogel polymers for microbial control in water systems benefits from significant inventions and technological advancements in related fields, which have yielded trends such as improved synthesis techniques that enhance the performance and properties of the polymers. However, a few areas remain understudied and warrant further investigation to fully harness the abilities of the polymers. These include the suitability of different hydrogel polymers for the elimination of specific types and species of microorganisms and cost comparisons of reuse versus the synthesis of new polymers.

## Figures and Tables

**Table 1:** Diameter of the inhibition zones of various hydrogel polymers against different bacteria.

Hydrogel Polymer	Bacteria	Diameter of Inhibition Zone (mm)	References
Poly(acrylamide) Hydrogel	*Escherichia coli*	13	[31]
Poly(vinyl alcohol) Hydrogel	*Staphylococcus aureus*	20	[32]
Poly(ethylene glycol) Hydrogel	*Pseudomonas aeruginosa*	18	[33]
Chitosan Hydrogel	*Bacillus subtilis*	22.4	[34]
Alginate Hydrogel	*Klebsiella pneumoniae*	14	[35]
Polyvinyl Alcohol/Carboxymethyl Cellulose (PVA/CMC) Hydrogel	*Proteus vulgaris*	19	[36]
WO3NPs-hydroxyethyl Cellulose Hydrogel	*Bacillus cereus*	17	[37]
Benzyl Isothiocyanate-β-cyclodextrin-κ-carrageenan (BITC- βCD-KC) Hydrogel	*Listeria monocytogenes*	16.7	[38]
Gelatin methacryloyl/Hyaluronic acid Hydrogel	*Enterococcus faecalis*	5.5	[39]
Hyaluronic Acid Hydrogel	*Escherichia coli*	20.7	[40]

## Data Availability

Data are contained within the article.
